# Supplementary material for: Seatbelts and raincoats, or banks and castles: Investigating the impact of vaccine metaphors
Source: PLoS One. 2024 Jan 3;19(1):e0294739. doi: 10.1371/journal.pone.0294739 (PMC10763949; doi:10.1371/journal.pone.0294739)
Supplement: Attachment — Submitted filename: Response to Reviewers.docx [file pone.0294739.s002.docx]

PONE-D-23-09747R1

Seatbelts and raincoats, or banks and castles: Investigating the impact of vaccine metaphors

PLOS ONE

Dear Dr. Flusberg,

Thank you for submitting your manuscript to PLOS ONE. After careful consideration, we feel that it has merit but does not fully meet PLOS ONE’s publication criteria as it currently stands. Therefore, we invite you to submit a revised version of the manuscript that addresses the points raised during the review process.

Thank you for improving the manuscript. In this round, two additional reviewers provided minor comments that should be fixed. On top of that, my comments are: (1) please use the singular form “between-subject” instead of “between-subjects,” (2) please provide p-values or at least the indication of the statistical significance of the differences in Table 4 (that was also asked by one of the Reviewers in the previous round), (3) when mentioning one-way ANOVA, please explicitly state what the factor (independent variable) is, (4) why you do not provide comparisons between two conditions that constitute your experimental design (i.e., metaphor vs. literal), as you declared (rows 188-189), (5) when reporting p-values on p. 20, please also report the means and test statistics.

***Thank you for this helpful summary. We have now made all the required changes to the manuscript and have addressed all five of these initial points:***

(1) ***We have updated all instances to the singular “between-subject.”***
(2) ***We have added a table legend and superscript symbols to Table 4 to indicate which ratings significantly differed within each cell, which corresponds to the results described in the main text.***
(3) ***We have clarified our one-way ANOVA variables in this section of the paper as well (Explicit Ratings of the Health Messages; page 21): “We conducted a series of one-way ANOVAs to compare the effect of each of the three stimulus vignettes for each of our five questions on ratings of understandability, informativeness, and persuasiveness.”***
(4) ***We provide comparisons between the Metaphor vs. Literal condition by including this factor in our VAM-pretest vs. VAM-posttest analysis (page 23). In this analysis, we collapse over the two possible metaphors for each question and thus address this critical factor. This is the central analysis where a comparison of these two conditions is warranted based on our original research question.***
(5) ***We have added all the means, SDs, and test statistics to this section of the results in the updated manuscript (pages 21-22).***

Please submit your revised manuscript by Oct 01 2023 11:59PM. If you will need more time than this to complete your revisions, please reply to this message or contact the journal office at plosone@plos.org. When you're ready to submit your revision, log on to <https://www.editorialmanager.com/pone/> and select the 'Submissions Needing Revision' folder to locate your manuscript file.

Please include the following items when submitting your revised manuscript:

- A rebuttal letter that responds to each point raised by the academic editor and reviewer(s). You should upload this letter as a separate file labeled 'Response to Reviewers'.
- A marked-up copy of your manuscript that highlights changes made to the original version. You should upload this as a separate file labeled 'Revised Manuscript with Track Changes'.
- An unmarked version of your revised paper without tracked changes. You should upload this as a separate file labeled 'Manuscript'.

If you would like to make changes to your financial disclosure, please include your updated statement in your cover letter. Guidelines for resubmitting your figure files are available below the reviewer comments at the end of this letter.

If applicable, we recommend that you deposit your laboratory protocols in [protocols.io](http://protocols.io/) to enhance the reproducibility of your results. Protocols.io assigns your protocol its own identifier (DOI) so that it can be cited independently in the future. For instructions see: <https://journals.plos.org/plosone/s/submission-guidelines#loc-laboratory-protocols>. Additionally, PLOS ONE offers an option for publishing peer-reviewed Lab Protocol articles, which describe protocols hosted on [protocols.io](http://protocols.io/). Read more information on sharing protocols at <https://plos.org/protocols?utm_medium=editorial-email&utm_source=authorletters&utm_campaign=protocols>.

We look forward to receiving your revised manuscript.

Kind regards,

Wojciech Trzebiński, Ph.D.
Academic Editor
PLOS ONE

Journal Requirements:

Please review your reference list to ensure that it is complete and correct. If you have cited papers that have been retracted, please include the rationale for doing so in the manuscript text, or remove these references and replace them with relevant current references. Any changes to the reference list should be mentioned in the rebuttal letter that accompanies your revised manuscript. If you need to cite a retracted article, indicate the article’s retracted status in the References list and also include a citation and full reference for the retraction notice.

[Note: HTML markup is below. Please do not edit.]

Reviewers' comments:

Reviewer's Responses to Questions

**Comments to the Author**

1. If the authors have adequately addressed your comments raised in a previous round of review and you feel that this manuscript is now acceptable for publication, you may indicate that here to bypass the “Comments to the Author” section, enter your conflict of interest statement in the “Confidential to Editor” section, and submit your "Accept" recommendation.

Reviewer #1: All comments have been addressed

Reviewer #3: All comments have been addressed

Reviewer #4: (No Response)

2. Is the manuscript technically sound, and do the data support the conclusions?

The manuscript must describe a technically sound piece of scientific research with data that supports the conclusions. Experiments must have been conducted rigorously, with appropriate controls, replication, and sample sizes. The conclusions must be drawn appropriately based on the data presented.

Reviewer #1: Yes

Reviewer #3: Yes

Reviewer #4: Yes

3. Has the statistical analysis been performed appropriately and rigorously?

Reviewer #1: Yes

Reviewer #3: Yes

Reviewer #4: Yes

4. Have the authors made all data underlying the findings in their manuscript fully available?

The [PLOS Data policy](http://www.plosone.org/static/policies.action#sharing) requires authors to make all data underlying the findings described in their manuscript fully available without restriction, with rare exception (please refer to the Data Availability Statement in the manuscript PDF file). The data should be provided as part of the manuscript or its supporting information, or deposited to a public repository. For example, in addition to summary statistics, the data points behind means, medians and variance measures should be available. If there are restrictions on publicly sharing data—e.g. participant privacy or use of data from a third party—those must be specified.

Reviewer #1: Yes

Reviewer #3: Yes

Reviewer #4: Yes

5. Is the manuscript presented in an intelligible fashion and written in standard English?

PLOS ONE does not copyedit accepted manuscripts, so the language in submitted articles must be clear, correct, and unambiguous. Any typographical or grammatical errors should be corrected at revision, so please note any specific errors here.

Reviewer #1: Yes

Reviewer #3: Yes

Reviewer #4: Yes

6. Review Comments to the Author

Please use the space provided to explain your answers to the questions above. You may also include additional comments for the author, including concerns about dual publication, research ethics, or publication ethics. (Please upload your review as an attachment if it exceeds 20,000 characters)

Reviewer #1: (No Response)

Reviewer #3: As a "second-round" reviewer I would like to acknowledge that the authors answered to the concerns and question raised by previous reviewers and add that: 1) (lines 156-157) literature on the impact of metaphorical framing on vaccine attitudes is not limited to Scherer and colleagues 2015 (see Ervas et al. 2022); 2) (question 4; metaphor 2 WAR) "With certain viruses, some people who get infected can experience" --> the word "can" is added, when compared to the other texts, with no apparent reason. In general, the texts (especially in the "literal version") are not well-balanced: the literal version is always shorter than the other versions and this might influence the ease and times for comprehension.

***Thank you for these excellent points. We were not aware of the Ervas et al. (2022) paper, which was published while we were working on our project. We have now included a brief discussion of this study in the introduction (page 9) and have added the paper to our references section.***

***We appreciate the observation that our stimuli may have been unbalanced on certain dimensions. However, we feel that our data suggest people processed them relatively similarly in certain respects: most of the explicit ratings of the stimulus texts were similar (Table 4) and all participants showed roughly the same improvement in vaccine attitudes from pre- to posttest, regardless of condition.***

***In addition to the ratings data and the lack of difference between conditions, it is worth noting that our claims that that the texts were balanced are also underpinned by the fact that we computed readability scores to ensure comparability of the texts based primarily on the work of Flesch and Flesch-Kincaid and others, using the tests here:*** [***https://readabilityformulas.com/free-readability-formula-tests.php***](https://readabilityformulas.com/free-readability-formula-tests.php)

***The website provides a free-to-use resource of popular readability measures to help gauge the readability or difficulty level of a text in English. We computed composite scores averaging seven popular readability measures to come up with a “Readability Consensus,” which we used to further establish comparability between our written texts. We have added information about this, and a reference to Flesch’s work, which we hope assuages the concern about comparability (page 14):***

***“We computed readability consensus scores to ensure comparability of the texts based primarily on the work of Flesch and Flesch-Kincaid and others [50], using a range of measures to gauge the readability or difficulty level of a text in English.”***

***Ultimately, our goal was to create more realistic messaging texts, which required slightly more prose in the metaphorical condition. We appreciate the point that there is an added “can” in the War metaphor stimulus, but, as we discuss in several places in the paper, that specific metaphor had more significant conceptual issues that limit its utility.***

Reviewer #4: This paper describes a very interesting study with a sound methodology and relevant findings for the examined field (which also provide useful insights into aspects of metaphor use that go beyond the main purpose of the study itself). For instance, some inputs are given as to which specific aspects of vaccination can be successfully explained through metaphor use (e.g., natural immunity vs vaccine immunity). Furthermore, the section with the free-text answers (with some metaphors used incorrectly but with the same conceptual mapping presented in the explanation stimulus, others used correctly but in the attempt to refute the statements presented in the explanation stimulus, others invented by respondents) is also an interesting starting point for future research.

***Thank you.***

The study limitations (e.g., resorting to only a selection of metaphors available in vaccine campaigns) and previous reviews’ comments (like some remarks concerning methodology, like why respondents needed to state how they thought a general audience would react to certain messages rather than express their own opinion directly – to avoid some sort of “self-censorship” due to the stigmatization of vaccine aversion and hesitancy in an emotive and politicized historical moment like the one right after the coronavirus pandemic) seem to have been addressed properly.
The conclusion is that it is deemed to be highly advisable to publish this paper.

***Thank you, we’re happy to see that our revision efforts successfully addressed the previous round of reviewer comments.***

Some (minor) remarks and/or suggestions that may help further improve the paper are the following:

ABSTRACT:
I would mention the denomination “literal responses” a line before. The current formulation may be initially confusing: while reading the abstract, my first thought was that the study was going to examine extended metaphors and non-extended ones (as defined later in the paper - in p. 8, line 167 - “one-off” metaphors). A possible solution would be: “We created three response passages for each question: two included extended explanatory metaphors and one contained a literal response, with no explanatory metaphors”).

***Thank you for highlighting this point of confusion in the abstract. We have taken your suggestion and updated the abstract accordingly in the revised manuscript.***

MAIN TEXT:
1) In the theoretical framework, when I read the section about extended explanatory metaphors (and their opposition to “one-off” metaphors) I thought about the frequent term “metaphor(ical) scenario” (as in Musolff 2006 or 2016*) with the “mininarratives”. It could be useful to add this terminology in the beginning to clarify what is meant in the text with “extended metaphors”. In case there is no full equivalence between this term and term “extended metaphors” as intended in the study, this could also be explained and motivated.
In addition, the term “metaphorical scenario” does indeed occur randomly in the paper (line 559, with respect to the Video Game metaphor, and line 574, with respect to the Raincoat metaphor), which further convinced me it could be useful to add it in the beginning, too.
N.B.: “scenario” also occurs a third time in line 583, in the segment “in the answers to the friend scenario”. As “scenario” is a technical term in metaphor studies, I would reformulate this part to avoid possible confusion.

***Thank you for this very useful suggestion. We have introduced and exemplified Musolff’s notion of scenario as part of the theoretical framework, and referred to it when introducing and discussing the extended metaphors used in the study (p. 5):***

***“Discourse analysts have, in some cases, found Lakoff and Johnson’s notion of the conceptual domain too broad to explain how metaphors are used in discourse, especially in the context of explanation and persuasion. In a series of studies on political discourse, Musolff [12,13] has employed the sub-domain notion of scenario as a “a set of assumptions made by competent members of a discourse community about ‘typical’ aspects of a source-situation, for example, its participants and their roles, the ‘dramatic’ storylines and outcomes, and conventional evaluations of whether they count as successful or unsuccessful, normal or abnormal, permissible or illegitimate, etc.” [12, p. 28]. In this way, the same overarching domain may generate different conceptual entailments under difference domain-related scenarios. For example, in a study of metaphors in cancer experience, Semino et al. [14] show how war-related metaphors can have different implications(e.g., for the (dis)empowerment of patients) depending on the specific scenario involved, such as PREPARING FOR BATTLE (e.g., “I am sharpening my weapons in case I have to do battle”) vs. OUTCOME OF BATTLE (e.g., “I’m not winning this battle ”). “***

2) I find that there are some similarities between some of the metaphors chosen for the study. More specifically, I feel that the Castle metaphor is a subtype of War metaphors (of course, with a different focus, but the mention of words like “invaders”, “defence” etc. seems to support this theory). I find this aspect important for several reasons. Firstly, on the basis of what is stated in the Linguistic Analysis at page 27: “[…] This is consistent with the finding that the War metaphor was perceived as particularly ineffective, as also shown in the ratings data described earlier. This is a particularly interesting finding given the prevalence of war messaging in vaccine discourse, particularly early on in the pandemic”. If the (more effective, “successful”) Castle metaphor is also regarded as a subtype of War metaphors, this once again shows that (although controversial!) war metaphors are quite effective, and this sentence should be modified accordingly.

To differentiate between these two metaphors, maybe a different (more specific, as occurs with Castle) label could be proposed for what is now called War metaphor in the paper survey (in Question 4, Metaphor 2), like Army Enrolment, Army Enlistment, or Mobilization.

Considering the Castle and Army Enrolment metaphors as subtypes belonging to the same source domain may also have an impact on the paper in other ways: as stated at page 24, some metaphors may be especially effective to communicate about some particular aspects of a phenomenon (e.g., Pilots and Fire Drills and a particular issue of natural immunity). If the categorization I put forward is implemented, the importance of choosing the right connection between a specific source and target (sub)domain is also better highlighted. So maybe war metaphors are effective in showing how vaccines work but not as effective in explaining the concept of herd immunity. This different effectiveness can also be explained through one of the variables reported in Table 1 at page 5 (army mobilization, unlike castle defence systems, is not very straightforward to many people! I also found this extended explanatory metaphor quite hard to follow as I first read it).

***Thank you for this important point. After extended discussion, we decided to keep the label War metaphor because the word ‘war’ occurs in the relevant stimulus text, in contrast with the Castle text. However, to address your comment, we have clarified throughout the paper that a specific scenario of mobilization is always involved. Concomitantly, we have revised our comments on the effectiveness of War metaphors accordingly. In the General Discussion, for example, we have explicitly pointed out the fact that both our Castle and War metaphors involve war-related scenarios and considered the implications of this for the significance of our findings using structural entailments in context as one key differentiating factor (pages 34-5):***

***“For example, both our quantitative and qualitative findings suggest that invoking a war metaphor involving a national mobilization scenario might be particularly ineffective in explaining why low-risk individuals should be vaccinated. Given that there is a wide range of possible available metaphors, using a different one is a simple choice. In contrast, the castle metaphor, which involves a different war-related scenario, was found to be potentially useful in communicating how vaccines work. This highlights the importance of considering specific scenarios and their associated narratives and structural entailments in developing metaphorical frames. Broader domains like WAR encompass a range of different scenarios that may be more or less effective when used as metaphors to explain particular target domains.”***

3) I think that the use of the rhetorical question “Would you rather wear a raincoat or walk around unprotected during a thunderstorm?” at page 31 should be highlighted, as it shows some sort of emotional involvement on part of the participant, which proves that this metaphor is effective.

***This is an excellent point. We have now added a sentence on page 33 to highlight this keen observation:***

***“In the latter example, the use of a rhetorical question and the contrast between wearing a raincoat and being ‘unprotected during a thunderstorm’ arguably have stronger evaluative and emotional associations than the wording used in the original stimulus text.”***

TYPOS:
p. 19, line 326: five response questions (I guess!)
p. 20, line 359: explicit
p. 25, line 452: abstract
p. 29, line 29: “[…] question asked was “How do vaccines work?”. The […]
p. 31, line 577: “raincoat even if” (or, if it was a mistake made by the participant, add [sic]!)
p. 32, line 597: missing full stop before “finally”
p. 32, line 604: (92106; 100% re-user) --> I think this parenthesis was not meant to be here, maybe it was a note/reference meant only for the authors which “slipped in” during writing. If this is not the case, it is not clear what this number refers to.
p. 36, line 688: there seems to be a missing space before “relatedly”
p. 37, line 711: after both occurrences of e.g. the comma is underlined

***Thank you for pointing us to these errors. We have corrected all of them in the updated manuscript.***

SOME POTENTIAL TYPOS/POINTS WITH POTENTIAL STYLISTIC IMPROVEMENTS:
Consider that I am not a native speaker of English, so these are some points that can be ignored by the authors if necessary:
p. 4, line 74: The seminal work […]?
p. 31, line 591: metaphor use? Or used?
p. 36, line 697: willingness/ the willingness to get vaccinated?

***We appreciate these suggestions and have incorporated them into the revised manuscript.***

OTHER TYPOGRAPHICAL INDICATIONS:
I recommend being more typographically consistent in the way metaphors are reported. For instance, in the theoretical framework some metaphors are written in italics (beast, riot etc., line 158). In line 697, the same metaphors have a standard font, with no italics. In the introduction, some metaphors are reported between inverted commas (e.g., the “Cake” metaphor, line 168), but then they have a standard font in other parts of the text (e.g., the Bank or Castle metaphor, line 362). Of course, small capitals can be kept as a way to refer to conceptualizations and domains (as occurs in the introduction at page 4, for instance).

***We appreciate this point. In the updated manuscript, we now use italics to indicate metaphorical language throughout the text, except when quoting directly from another source or a participant free-response.***

Line 662: seven in digits, maybe?

***We have changed the 10 to “ten” in this instance to adhere with general formatting preferences for numbers below 11.***

Use of hyphens: in some cases (like page 8, line 162-3, or 184-5) hyphens are longer and words are not separated by a space. In other cases, like at page 34 (line 658) the hyphen is shorter and there is a space that separates it from the words.

***We now use the longer “em dash” with no extra spaces throughout the text.***

Pag. 18, line 313: maybe here the usual indication “[…]” should be used instead of the three dots?

***We have made this update to the revised manuscript***

*Musolff A. (2006) “Metaphor scenarios in public discourse”, Metaphor and Symbol, 21:1, pp. 23-38.
Musolff A. (2016) Political metaphor analysis: Discourse and scenarios, Bloomsbury Academic.

7. PLOS authors have the option to publish the peer review history of their article ([what does this mean?](https://journals.plos.org/plosone/s/editorial-and-peer-review-process#loc-peer-review-history)). If published, this will include your full peer review and any attached files.


If you choose “no”, your identity will remain anonymous but your review may still be made public.


**Do you want your identity to be public for this peer review?** For information about this choice, including consent withdrawal, please see our [Privacy Policy](https://www.plos.org/privacy-policy).

Reviewer #1: **Yes:** Amanda J Chase

Reviewer #3: No

Reviewer #4: No

[NOTE: If reviewer comments were submitted as an attachment file, they will be attached to this email and accessible via the submission site. Please log into your account, locate the manuscript record, and check for the action link "View Attachments". If this link does not appear, there are no attachment files.]

While revising your submission, please upload your figure files to the Preflight Analysis and Conversion Engine (PACE) digital diagnostic tool, <https://pacev2.apexcovantage.com/>. PACE helps ensure that figures meet PLOSrequirements. To use PACE, you must first register as a user. Registration is free. Then, login and navigate to the UPLOAD tab, where you will find detailed instructions on how to use the tool. If you encounter any issues or have any questions when using PACE, please email PLOS at figures@plos.org. Please note that Supporting Information files do not need this step.
