# Supplementary material for: Seatbelts and raincoats, or banks and castles: Investigating the impact of vaccine metaphors
Source: PLoS One. 2024 Jan 3;19(1):e0294739. doi: 10.1371/journal.pone.0294739 (PMC10763949; doi:10.1371/journal.pone.0294739)
Supplement: Attachment — Submitted filename: Response to Reviewers.docx [file pone.0294739.s003.docx]

PONE-D-23-09747R2

Seatbelts and raincoats, or banks and castles: Investigating the impact of vaccine metaphors

PLOS ONE

Dear Dr. Flusberg,

Thank you for submitting your manuscript to PLOS ONE. After careful consideration, we feel that it has merit but does not fully meet PLOS ONE’s publication criteria as it currently stands. Therefore, we invite you to submit a revised version of the manuscript that addresses the points raised during the review process.

Please submit your revised manuscript by Dec 02 2023 11:59PM. If you will need more time than this to complete your revisions, please reply to this message or contact the journal office at [plosone@plos.org](mailto:plosone@plos.org). When you're ready to submit your revision, log on to <https://www.editorialmanager.com/pone/> and select the 'Submissions Needing Revision' folder to locate your manuscript file.

Please include the following items when submitting your revised manuscript:

- A rebuttal letter that responds to each point raised by the academic editor and reviewer(s). You should upload this letter as a separate file labeled 'Response to Reviewers'.
- A marked-up copy of your manuscript that highlights changes made to the original version. You should upload this as a separate file labeled 'Revised Manuscript with Track Changes'.
- An unmarked version of your revised paper without tracked changes. You should upload this as a separate file labeled 'Manuscript'.

If you would like to make changes to your financial disclosure, please include your updated statement in your cover letter. Guidelines for resubmitting your figure files are available below the reviewer comments at the end of this letter.

If applicable, we recommend that you deposit your laboratory protocols in [protocols.io](http://protocols.io) to enhance the reproducibility of your results. Protocols.io assigns your protocol its own identifier (DOI) so that it can be cited independently in the future. For instructions see: <https://journals.plos.org/plosone/s/submission-guidelines#loc-laboratory-protocols>. Additionally, PLOS ONE offers an option for publishing peer-reviewed Lab Protocol articles, which describe protocols hosted on [protocols.io](http://protocols.io). Read more information on sharing protocols at <https://plos.org/protocols?utm_medium=editorial-email&utm_source=authorletters&utm_campaign=protocols>.

We look forward to receiving your revised manuscript.

Kind regards,

Wojciech Trzebiński, Ph.D.
Academic Editor
PLOS ONE

Journal Requirements:

Please review your reference list to ensure that it is complete and correct. If you have cited papers that have been retracted, please include the rationale for doing so in the manuscript text, or remove these references and replace them with relevant current references. Any changes to the reference list should be mentioned in the rebuttal letter that accompanies your revised manuscript. If you need to cite a retracted article, indicate the article’s retracted status in the References list and also include a citation and full reference for the retraction notice.

Additional Editor Comments:

I appreciate your improvements, and the reviewers accept your revised manuscript. However, I have one more crucial doubt that needs to be addressed before the acceptance. Namely, one may understand from your Abstract that the only positive result you have consistently reached is that “participants in the metaphor condition provided longer free-response answers to the question posed by a hypothetical friend” (rows 39-41). If so, it is crucial to provide some argument that this difference is statistically significant. In Table 5, you report the “cumulative” word count. If it is a sum of words used by all participants in particular conditions, it depends on the sample sizes. So, it would probably be necessary to calculate the means of the word count per condition. Then, you should compare the means between conditions to check the statistical significance.

**We appreciate and agree with this suggestion. We have now conducted the appropriate calculations and statistical tests, which we have incorporated into the updated manuscript (pages 27-8). The inferential statistics provide further support for the conclusion presented in the abstract and general discussion: participants in the metaphor condition used significantly more words on average (~18% overall) compared to participants in the literal condition in response to the free text “explain to a friend” prompt across all the questions.**

**We have expanded Table 5 in this section of the paper to provide more descriptive data about the mean word counts for each question and condition. All our in-text discussion of these findings, as well as a revised Figure 3, now present the mean rather than cumulative word count data. We feel that presenting the data in this way and making these changes has strengthened this section of the paper as well as our overall conclusions.**

**Of note, while conducting these new analyses we discovered a single numerical error in our earlier manuscript. Specifically, we had under-reported the cumulative word count in the free text response for the *war* metaphor passage in response to Question 4. This appears to have been a random transcription error as we compiled the original manuscript, as an early version of the table we created included the correct number. This error is minor and only served to weaken our original conclusions. We have corrected the error and modified our discussion accordingly. We now have even stronger evidence to support our conclusion that participants who received a metaphorical passage used more words in their “explain to a friend” free text responses.**

**Considering all this, however, we took this opportunity to double check all the analyses and values reported throughout the paper (in figures and text) to ensure accuracy. We discovered no other errors in the manuscript, so we are confident in the accuracy of our reporting. In the spirit of transparency, our raw data is accessible via an updated, now public link to the Open Science Framework.**

Please also double-check the manuscript for typos (e.g., row 566).

**We have now double-checked the manuscript for typos and other typographical errors and believe everything is in order.**

6. Review Comments to the Author

Please use the space provided to explain your answers to the questions above. You may also include additional comments for the author, including concerns about dual publication, research ethics, or publication ethics. (Please upload your review as an attachment if it exceeds 20,000 characters)

Reviewer #3: The authors addressed all my questions/concerns. Thanks for the opportunity to read this piece of research.

Reviewer #4: At this stage of review, I only want to point out a few typos/stylistic suggestions:

War mobilization in italics, lines 526-7

line 632: and not capitalized

line 619-620: and not capitalized twice

We return to this finding (lines 470, 544): I think this could be formulated in a better way stylistically, like "This finding will be further discussed/discussed in greater detail ...etc. "

But see 37 (line 670): I find this could also be expressed in a better way

Posttest is sometimes written with an hyphen, sometimes without it; I do not understand if there is a reason to it

**We have addressed all these issues in the revised manuscript.**
